# Cytogenetic contribution to uniparental disomy (UPD)

**DOI:** 10.1186/1755-8166-3-8

**Published:** 2010-03-29

**Authors:** Thomas Liehr

**Affiliations:** 1 Jena University Hospital, Institute of Human Genetics and Anthropology, Kollegiengasse 10, D-07743 Jena, Germany

## Abstract

Uniparental disomy (UPD) is often considered as an event to be characterized exclusively by molecular genetic or epigenetic approaches. This review shows that at least one third of UPD cases emerge in connection with or due to a chromosomal rearrangement. Thus, additional (molecular) cytogenetic characterization of UPD cases is essential. Up to now > 1,100 UPD cases detected in clinical, non-tumor cases are reported in the literature. Recently, these cases were summarized in a regularly updated, freely available online database http://www.med.uni-jena.de/fish/sSMC/00START-UPD.htm. Based of this, here the presently known imprinting syndromes, the chromosomal contribution to UPD phenomenon, and the cytogenetic subgroups of UPD, including cases with normal, abnormal balanced or unbalanced karyotype (like e.g. small supernumerary marker chromosomes and Robertsonian translocations) and segmental UPD are reviewed. Furthermore, chromosome fragmentation as a possible mechanism of trisomic rescue is discussed, which might help to explain the observed 1:9 rate of maternal versus paternal UPD present in cases with original trisomic karyotypes. Overall, as UPD is more but an interesting rarity, the genetic background of each "UPD-patient" needs to be characterized besides by molecular methods, also by molecular cytogenetics in detail.

## Introduction

Uniparental disomy (UPD) is the presence of a chromosome pair derived only from one parent present in a disomic cell line [1]. When one of the first proven UPD case was published [2] an editorial in the same journal issue commented this by the words: < it seems unlikely that UPD will turn out to be anything but an interesting rarity > [3]. However, today, some 20 years later, there are > 1,100 reports on UPD cases [1] and what was considered initially as something exotic is nowadays an important diagnostic [4] and even prognostic factor for special syndromes [5,6]. Also UPD is able to support the localization of monogenic disorder genes (e.g. [7], see also [1]) and was demonstrated to play a role in tumorigenesis, as reviewed by [8].

The concept of UPD was introduced in 1980 into medical genetics by Eric Engel [9]. In 1987 the first case of UPD proven by molecular methods was described [10]. However, cases having a UPD were reported before [11-14].

In theory there are 48 possible uniparental chromosomal pairs, plus 2 whole genomic variants of UPD which could exist. Up to present no maternal UPD was reported for chromosome 19 (and Y), and no paternal UPD for chromosomes 4, 17, 18 and 19 [1]. UPD can be detected based on cytogenetic data and chromosomal heteromorphisms or rearrangements [10-14], microsatellite analysis [15], methylation test [16] or SNP-bases array-comparative genomic hybridization [15]. Also molecular cytogenetics taking advantage of the so called copy number variations (CNV) within the human genome can be used to characterize UPD [17]. Interestingly, UPD is in at least 30% of the case observed together with a chromosomal aberration [1]. Thus, (molecular) cytogenetics is essential when concentrating on this putatively exclusive molecular genetic topic.

This review focuses on UPD present in clinically normal and clinically abnormal persons. UPD cases nowadays repeatedly reported as acquired, tumor-specific epigenetic alteration [8] are not subject of this paper. Basis of this review is a freely available and regularly updated online database including all published UPD-cases [1].

## Basic types of UPD

According to [18] there are three basis types of UPD. UPD for the entire chromosomal complement can be present as paternal or maternal UPD (UPDpat/UPDmat). The first leads to complete hydatidiform mole, the second one induces benign cystic ovary. For UPDpat exceptional cases are reported with mosaic state of uniparental and biparental inheritance but no triploidy; more frequently UPDpat cell lines with a triploid one are seen as partial hydatidiform mole [1,18].

UPD for a complete chromosome can appear due to gamete complementation, trisomic rescue (with or without formation of a small supernumerary marker chromosome = sSMC), monosomic rescue, mitotic error in connection with a Robertsonian or other translocation, isochromosome formation, deletion and duplication [18]. Finally, segmental UPD can arise due to a postzygotic somatic recombination between maternal and paternal homologue, or in connection with numerical and/or structural chromosomal aberrations [18].

## Hetero- and isodisomy, imprinting and hemizygosity

Two subtypes of UPD can be recognized by molecular analysis. The one is called heterodisomy (hUPD) and is defined as inheritance of both chromosomes from one parental pair. Besides there can be isodisomy (iUPD), i.e. inheritance of two copies of the same chromosomes from one parent. hUPD and iUPD can cause disease if affecting a gene underlying genomic imprinting (= expression of a gene which depends on parental origin). iUPD can further and independent of imprinting, result in functional reduction to hemizygosity and thus can cause a recessive disease to occur in the offspring of one carrier patient. Apart from monosomic rescue cases, which should always be iUPD, hUPD and iUPD can be observed as mixed forms mostly. Overall, mainly meiotic I or II errors and/or postzygotic events contribute to UPD formation [18]. Additionally, as Albert Schinzel stated < the incidence of meiotic nondisjunction increases with advanced maternal age, maternal UPD most often is heterodisomy while in paternal UPD isodisomy prevails, and no correlation with paternal age is found > [19].

As "imprinting disorders" are nowadays regarded and registered in the database Online Mendelian Inheritance of Man (OMIM) [20]:

- patUPD(6): transient neonatal diabetes (TND; OMIM #601410),

- matUPD(7): Silver Russel syndrome (SRS; OMIM #180860),

- patUPD(11): Beckwith-Wiedemann syndrome (BWS; OMIM #130650),

- matUPD(14): Temple syndrome (TS; see OMIM *605636 and #176270),

- patUPD(14): paternal UPD(14) syndrome (patUPD(14); OMIM #608149),

- matUPD(15): Prader Willi syndrome (PWS; OMIM #176270), and

- patUPD(15): Angelman syndrome (AS; OMIM #105830).

Meiotic and mitotic origin of the UPD has been shown to be assorted in different imprinting disorders. Meiotic origin is e.g. suggested in 58% of matUPD(7)-, in 89% of matUPD(15)- and in 16% in patUPD(15)- cases [21].

## Frequency of UPD

According to the literature the frequency of UPD in newborn is considered to be about 1 in 3,500 which equals a rate of 0.029% [21]. However, the rate for a similar rare human group of disorders, in which UPD also can be present, i.e. the patients with small supernumerary marker chromosomes (sSMC), is only 0.044% in newborn [22]. At present almost 4,000 sSMC cases [23] and only ~1,100 UPD [1] cases are reported in the literature. Thus, the rate of UPD in human population might be even lower than suggested, maybe 1 in 5,000 or less.

However, in the above mentioned "imprinting disorders" the UPD rates are much higher. In SRS about 5% of the cases show a matUPD(7). For AS a UPD rate of 7% [24] and for PWS of 25% is given [24,25]. BWS has segmental UPD(11p) in 20% of the cases. In TND a patUPD(6) is reported in 40% of the patients. > 95% of TS cases show matUPD(14) and for patUPD(14) syndrome no cases without UPD are reported yet [24]. Also important to state is that UPD seems not to be promoted by assisted reproductive technologies (ART), while imprinting defects are very well found more frequently after application of ART [24].

As summarized in Table 1 the male to female ratio of UPD carriers overall is 1:1. Only for matUPD(4) and patUPD(6) this ratio was abnormal (Tab. 1). For matUPD(4) at present eight cases are reported, seven of which are male. In patUPD(6) only seven of the nineteen cases are female. Further cases have to be studied to be able to draw a final conclusion if these data are subject to bias or not.

**Table 1:** Male to female ratio in maternal and paternal UPD for human autosomes.

	matUPD		patUPD	
	
Chromosome	Male	female	male	female
**1**	6	5	6	9

**2**	9	7	6	4

**3**	1	4	0	0

**4**	7	1	0	0

**5**	0	1	1	1

**6**	3	3	12	7

**7**	17	20	6	3

**8**	2	3	1	1

**9**	4	8	0	0

**10**	3	1	0	0

**11**	2	1	7	8

**12**	1	3	0	1

**13**	2	3	3	3

**14**	20	24	12	15

**15**	36	25	23	23

**16**	13	13	0	2

**17**	1	2	0	0

**18**	0	1	0	0

**19**	0	0	0	0

**20**	3	2	1	0

**21**	3	1	1	3

**22**	3	5	2	1

**Overall**	136	133	81	81

As not for all reported UPD cases the gender is given in the literature only 413 cases summarized in [1] could be included in this table.

## Detection of UPD

In one third of clinical UPD cases this genetic defect is uncovered due to, or in connection with a chromosomal abnormality [1]. In such cases cytogenetic analysis was performed because in the overwhelming majority of the cases with a UPD clinical abnormalities are present. Only some 50 cases with UPD but no clinical abnormalities are reported in the literature; they were found more or less by chance or due to repeated abortions in a family with chromosomal rearrangement [1].

**Figure 1:**
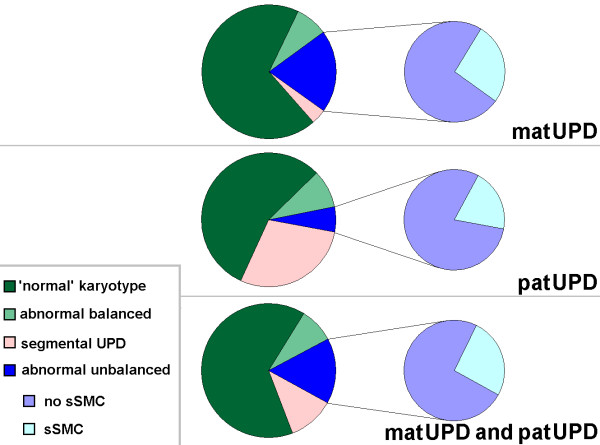
**Cytogenetic subgroups displaying matUPD, patUPD and UPD in general**. Abbreviations: matUPD = maternal UPD; patUPD = paternal UPD.

As the karyotype is crucial for the understanding of UPD formation, in the following the reported UPD-cases are grouped into: 1) Cases with proven or suggested normal karyotype; 2) cases with abnormal balanced karyotype; 3) cases with abnormal unbalanced karyotype, subdividing in a) cases with small supernumerary marker chromosomes (sSMC) and b) cases with imbalances except for sSMC; and 4) cases with segmental UPD. The percentage rates given below are summarized in Fig. 1 and deduced from [1].

## Cytogenetic subgroups displaying UPD

### 1) UPD-cases with proven or suggested normal karyotype

65% of the reported UPD cases have a cytogenetically proven or suggested normal karyotype of 46, XX or 46, XY (Fig. 1). Surprisingly, > 50% of these cases are published without having cytogenetics done. This is especially critical in UPD of one of the chromosomes 13, 14, 15, 21 or 22 (acrocentric ones). Presence of a Robertsonian translocation is known to contribute to the formation of this rare kind of mitotic error and can assist to explain familial cases of e.g. PWS or AS [26]. Between 0.6% and 3% of Robertsonian translocations are associated with UPD [27]. However, over 10% of the acrocentric chromosome derived UPDs summarized in [1] have a Robertsonian translocation.

### 2) UPD-cases with abnormal balanced karyotype

Abnormal balanced karyotypes in connection with UPD can be formed as isochromosomes (as described for chromosomes 1, 2, 4, 7 and 9), inversions (reported for chromosomes 3 and 4), balanced translocations (see chromosomes 7, 15) and Robertsonian translocations (chromosomes 13, 14, 15, 21 and 22). Overall, such abnormal balanced karyotypes are reported in 8% of published UPD cases [1] (Fig. 1).

### 3) UPD-cases with abnormal unbalanced karyotype

16% of reported UPD cases are observed in connection with an unbalanced karyotype. About a quarter of those cases were identified due to the presence of an sSMC [1,23] (Fig. 1).

#### 3a) UPD-cases with sSMC

All reported sSMC cases are summarized at http://www.med.uni-jena.de/fish/sSMC/00START.htm[23]. At present there are > 3,900 entries with sSMC; ~3,300 of those are reported not in connection with a Turner syndrome. Thus, at least 1.3% of those sSMC cases present with a UPD. This rate would be higher if derivative-chromosome-22-/Emanuel-syndrome would be included; however, to the best of our knowledge no reliable quantitative data on parental UPD(22) origin is available here. Among the reported UPD cases sSMC were present in 4%. As up to present only de novo sSMC were associated with a UPD, trisomic rescue is the most likely reason for their formation. This assumption was also already proven for some UPD cases (e.g. [28]).

#### 3b) UPD-cases with imbalances except for sSMC

Trisomic rescue can not only lead to sSMC formation but also, more often, to mosaic formation like mos 47, XN, + 16/46, XN; in such cases a UPD, in the given example a UPD 16, can be present in the cells with normal karyotype. Over 100 such cases are reported, at present for chromosomes 2, 4, 6-7, 9-17 and 20-22. Besides, imbalances like additional sex chromosomes, pseudodicentric chromosomes, unbalanced translocations, partial deletions and duplications are reported in the remainder ~25 cases. In UPD-cases with additional imbalances it is hard to distinguish between a possible effect of UPD and of the observed chromosomal imbalance on the clinical phenotype. Also, if no chromosomal imbalance has been detected except for UPD, there still can be aberrant cell lines present in other than the studied body tissues. This topic is matter of discussion especially for matUPD(16) [29]. Additionally, it is noteworthy that in the here discussed mosaic cases with a trisomic cell line the disomic cells have a UPD and the trisomic ones not. However, there are mosaic cases with UPD reported especially for cases with BWS, but also other chromosomes [1]. Mostly these can be acknowledged only by molecular approaches, as cytogenetically the different cell lines do not differ.

### 4) Segmental UPD-cases

At present there are 122 reports on segmental UPD. ~65% of those cases are provided by Beckwith-Widemann syndrome and segmental paternal UPD 11p [1]. The remainder cases were found in connection with chromosomal rearrangements in ~12%, while a normal or no karyotype is reported in 20 and 16 cases, respectively. Overall, 11% of all known UPD cases are of the segmental type (Fig. 1).

## Chromosomal contribution to UPD in autosomal chromosomes

**Figure 2:**
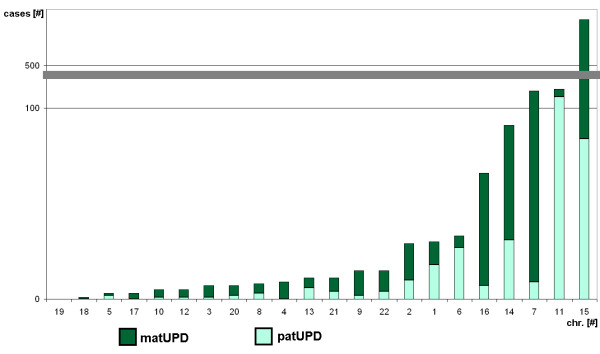
**Chromosomal contribution to UPD**. Maternal (matUPD) and paternal (patUPD) are presented in different colors. Abbreviations: # = number; chr. = chromosome.

In Fig. 2 the chromosomal contribution to UPD together with matUPD and patUPD is summarized. Chromosomes 15, 11, 7, 14 and 16 are most often involved in UPD formation, chromosome 15 being by far the most often observed one, which might possibly reflect in parts an ascertainment bias. Chromosomes 1, 2 and 6 have a moderate frequency of UPD, while the remainder chromosomes are sparsely contributing to UPD.

**Figure 3:**
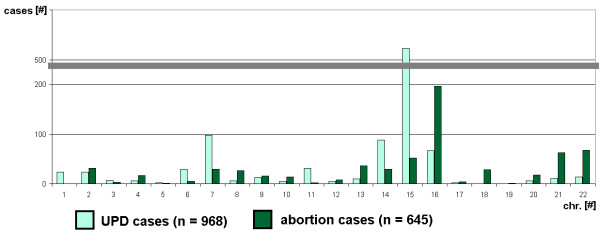
**Chromosomal distribution of UPD compared to that found in abortions**. Abbreviations: # = number; chr. = chromosome; n = quantity of cases.

Kalouseck and Barrett [30] suggested that the frequency with which various chromosomes are involved in placental aneuploidy could correspond to the incidence of specific chromosomal trisomies in spontaneous abortions. Thus, a data from a review on chromosomal contribution in spontaneous abortion [31] was compared with chromosomal contribution of UPD as known by now, excluding segmental UPD cases [1]. However, as visible in Fig. 3 there is no positive correlation, but, if at all a negative one. If true, this would possibly mean that chromosomes which tend to form UPD are found less frequently in abortions. Further studies are required to substantiate this possibility.

## Maternal and paternal UPD

Trisomic rescue as reason for UPD is present in most of the sSMC- and of the mosaic-cases having trisomy in a subset of their cells. Overall, about 150 such cases are available in the literature. The fact that only 4 of 45 sSMC- and 9 of 107 mosaic-carriers show a paternal UPD, i.e. only 8.6%, must have a biological background. It was already speculated that this might be due to the higher frequency of aneuploidy in oocytes than in sperm [32]. Chromosome anomalies are common in human gametes, with approximately 21% of oocytes and 9% in sperm [33]. In implanted embryos the rate of trisomies was estimated to be 16% [34]. Trisomic rescue is the result of viable postzygotic non-disjunction or anaphase lag event occurring during early embryogenesis which can involve either trophectoderm or extraembryonic mesoderm progenitors or both of them [35]. Los and coworkers added to that in 1998 the theory of chromosome demolition as an alternative correction mode [36]. As UPD and sSMC formation can go together, chromosome demolition would be a process of deliberate fragmentation and/or removal of one of the sets of three chromosomes during ana- or metaphase. Such chromosome fragmentation is seen in Howel Joly bodies [37] and a case with a del(5)(q31) recently reported could be interpreted as incomplete chromosome fragmentation [38]. Also the recently recognized phenomenon that "developmental chromosome instability" is significantly increased during embryonic stage and affects different tissues is to be mentioned in this context [39]. Los and coworkers [36] < consider trisomic rescue to consist of one correction event in the first to fourth postzygotic cell division with a subsequent unknown distribution of trisomic and disomic cells among the progenitor cells of the inner cell mass and trophoblast compartment until 16-cell stage >. Cellular selection during following formation of placenta and early embryogenesis would help as a result to ensure the presence of a numerically balanced chromosome complement in the developing fetus.

Together with the recent findings that there are inherent epigenetic differences between the paternal and maternal pronuclei in early cleavage stage embryos [40] this led us to suggest the following idea to explain the above mentioned 1:9 rate of matUPD versus patUPD. Besides the fact that aneusomies are more likely to be contributed from the female side [32] also another kind of enzymatic content in male and female derived pronuclear compartment could be important. The oocyte has obviously a less active machinery to eliminate chromosomal mistakes than the spermatocyte. Thus, at stage of pronuclei an elimination of a paternally derived additional chromosome could be more likely than of a maternally derived one. In concordance herewith, evidence for the existence of a chromosome counting mechanisms in zygote and early embryogenesis was already provided [41].

## Conclusion

Based on a regularly updated case collection on clinical UPD cases [1] meta-analyses of the presently available case-reports are now possible. Here a first attempt is presented. Similar as stated for the sSMC database [23] in [42] the present UPD-cases collected [1] are ascertainment-biased, however, it is the only data available by now. Of the 48 possible uniparental chromosomal pairs, plus 2 whole genomic variants of UPD still the first reports for mat UPD(19) and patUPD(4), patUPD(17), patUPD(18), and patUPD(19) are awaited. However, the rate of UPD might be lower than that of sSMC, and thus, lower than predicted [21]. Trisomic rescue and UPD should be highly actual fields of research, as the understanding of nuclear architecture and interphase cell regulation is nowadays considered as important for epigenetic gene regulation [43]. Thus, interphase cytogenetic tools like fluorescence in situ hybridization based banding approaches including multicolor banding could also be of interest in UPD-research [43-46].

What cannot be stressed enough is that the genetic background of a 'UPD-patient' needs to be characterized, besides by molecular methods, also by (molecular) cytogenetics as in one third of the cases chromosomal rearrangements are in connection with the event of a UPD!

## Competing interests

The author declares that he has no competing interests.

## Authors' contributions

TL contributed all work related to the UPD-database [1] and to this review.

## References

[B1] Liehr T Cases with uniparental disomy (UPD) 2010 http://www.med.uni-jena.de/fish/sSMC/00START-UPD.htm 10.1186/1755-8166-3-8 PMC2853554 20350319

[B2] Spence JE Perciaccante RG Greig GM Willard HF Ledbetter DH Hejtmancik JF Pollack MS O'Brien WE Beaudet AL Uniparental disomy as a mechanism for human genetic disease Am J Hum Genet 1988 42 217 226 2893543 PMC1715272

[B3] Warburton D Uniparental disomy: a rare consequence of the high rate of aneuploidy in human gametes Am J Hum Genet 1988 42 215 216 3341379 PMC1715263

[B4] Eggermann T Meyer E Ranke MB Holder M Spranger S Zerres K Wollmann HA Diagnostic proceeding in Silver-Russell syndrome Mol Diagn 2005 9 205 209 10.2165/00066982-200509040-00007 16392900

[B5] Halit H Grice SJ Bolton R Johnson MH Face and gaze processing in Prader-Willi syndrome J Neuropsychol 2008 2 65 77 10.1348/174866407X243305 19334305

[B6] Weksberg R Squire JA Molecular biology of Beckwith-Wiedemann syndrome Med Pediatr Oncol 1996 27 462 469 10.1002/(SICI)1096-911X(199611)27:5<462::AID-MPO13>3.0.CO;2-C 8827075

[B7] Woodage T Prasad M Dixon JW Selby RE Romain DR Columbano-Green LM Graham D Rogan PK Seip JR Smith A Bloom syndrome and maternal uniparental disomy for chromosome 15 Am J Hum Genet 1994 55 74 80 7912890 PMC1918231

[B8] Tuna M Knuutila S Mills GB Uniparental disomy in cancer Trends Mol Med 2009 15 120 128 10.1016/j.molmed.2009.01.005 19246245

[B9] Engel E A new genetic concept: uniparental disomy and its potential effect, isodisomy Am J Med Genet 1980 6 137 143 10.1002/ajmg.1320060207 7192492

[B10] Créau-Goldberg N Gegonne A Delabar J Cochet C Cabanis MO Stehelin D Turleau C de Grouchy J Maternal origin of a de novo balanced t(21q21q) identified by ets-2 polymorphism Hum Genet 1987 76 396 398 10.1007/BF00272452 2886422

[B11] Carpenter NJ Say B Barber ND A homozygote for pericentric inversion of chromosome 4 J Med Genet 1982 19 469 471 10.1136/jmg.19.6.469 6185681 PMC1048967

[B12] Kirkels VG Hustinx TW Scheres JM Habitual abortion and translocation (22q;22q): unexpected transmission from a mother to her phenotypically normal daughter Clin Genet 1980 18 456 461 7449187 10.1111/j.1399-0004.1980.tb01794.x

[B13] Palmer CG Schwartz S Hodes ME Transmission of a balanced homologous t(22q;22q) translocation from mother to normal daughter Clin Genet 1980 17 418 422 7398113 10.1111/j.1399-0004.1980.tb00173.x

[B14] Betz A Turleau C de Grouchy J Heterozygosity and homozygosity for a pericentric inversion of human chromosome 3 Ann Genet 1974 17 79 80 4547944

[B15] Altug-Teber O Dufke A Poths S Mau-Holzmann UA Bastepe M Colleaux L Cormier-Daire V Eggermann T Gillessen-Kaesbach G Bonin M Riess O A rapid microarray based whole genome analysis for detection of uniparental disomy Hum Mutat 2005 26 153 159 10.1002/humu.20198 15968682

[B16] Baumer A Wiedemann U Hergersberg M Schinzel A A novel MSP/DHPLC method for the investigation of the methylation status of imprinted genes enables the molecular detection of low cell mosaicisms Hum Mutat 2001 17 423 430 10.1002/humu.1118 11317358

[B17] Mkrtchyan H Gross M Hinreiner S Polytiko A Manvelyan M Mrasek K Kosyakova N Ewers E Nelle H Liehr T Volleth M Weise A Early embryonic chromosome instability results in stable mosaic pattern in human tissues PLoS One 2010 5 e9591 10.1371/journal.pone.0009591 20231887 PMC2834743

[B18] Gardner RJM Sutherland GR Chromosome abnormalities and genetic counseling 2004 Oxford University Press 314 318

[B19] Schinzel A Catalogue of unbalanced chromosome aberrations in man De Gruyter 2001 19 22

[B20] Online Mendelian Inheritance of Man (OMIM) 2010 http://www.ncbi.nlm.nih.gov/omim

[B21] Robinson WP Mechanisms leading to uniparental disomy and their clinical consequences Bioessays 2000 22 452 459 10.1002/(SICI)1521-1878(200005)22:5<452::AID-BIES7>3.0.CO;2-K 10797485

[B22] Liehr T Weise A Frequency of small supernumerary marker chromosomes in prenatal, newborn, developmentally retarded and infertility diagnostics Int J Mol Med 2007 19 719 731 17390076

[B23] Liehr T Small supernumerary marker chromosomes (sSMC) 2010 http://www.med.uni-jena.de/fish/sSMC/00START.htm 10.1159/000079572 15305057

[B24] Amor DJ Halliday J A review of known imprinting syndromes and their association with assisted reproduction technologies Hum Reprod 2008 23 2826 2834 10.1093/humrep/den310 18703582

[B25] Nicholls RD Knoll JH Butler Karam S Lalande M Genetic imprinting suggested by maternal heterodisomy in nondeletion Prader-Willi syndrome Nature 1989 342 281 285 10.1038/342281a0 2812027 PMC6706849

[B26] Flori E Biancalana V Girard-Lemaire F Favre R Flori J Doray B Mandel JL Difficulties of genetic counseling and prenatal diagnosis in a consanguineous couple segregating for the same translocation (14;15) (q11;q13) and at risk for Prader-Willi and Angelman syndromes Eur J Hum Genet 2004 12 181 186 10.1038/sj.ejhg.5201134 14694357

[B27] Ruggeri A Dulcetti F Miozzo M Grati FR Grimi B Bellato S Natacci F Maggi F Simoni G Prenatal search for UPD 14 and UPD 15 in 83 cases of familial and de novo heterologous Robertsonian translocations Prenat Diagn 2004 24 997 1000 10.1002/pd.961 15614836

[B28] Bartels I Schlueter G Liehr T Von Eggeling F Starke H Glaubitz R Burfeind P Supernumerary small marker chromosome (SMC) and uniparental disomy 22 in a child with confined placental mosaicism of trisomy 22: Trisomy rescue due to marker chromosome formation Cytogenet Genome Res 2003 101 103 105 10.1159/000074163 14610348

[B29] Wolstenholme J An audit of trisomy 16 in man Prenat Diagn 1995 15 109 121 10.1002/pd.1970150202 7784361

[B30] Kalousek DK Barrett I Confined placental mosaicism and stillbirth Pediatr Pathol 1994 14 151 159 10.3109/15513819409022034 8159612

[B31] Hassold T Chen N Funkhouser J Jooss T Manuel B Matsuura J Matsuyama A Wilson C Yamane JA Jacobs PA A cytogenetic study of 1000 spontaneous abortions Ann Hum Genet 1980 44 151 178 10.1111/j.1469-1809.1980.tb00955.x 7316468

[B32] Bán Z Nagy B Papp C Beke A Tóth-Pál E Papp Z Recurrent trisomy 21 and uniparental disomy 21 in a family Fetal Diagn Ther 2003 18 454 458 10.1159/000073142 14564119

[B33] Martin RH Meiotic errors in human oogenesis and spermatogenesis Reprod Biomed Online 2008 16 523 531 18413061 10.1016/s1472-6483(10)60459-2

[B34] Farfalli VI Magli MC Ferraretti AP Gianaroli L Role of aneuploidy on embryo implantation Gynecol Obstet Invest 2007 64 161 165 10.1159/000101741 17934313

[B35] Kalousek DK Howard-Peebles PN Olson SB Barrett IJ Dorfmann A Black SH Schulman JD Wilson RD Confirmation of CVS mosaicism in term placentae and high frequency of intrauterine growth retardation association with confined placental mosaicism Prenat Diagn 1991 11 743 750 10.1002/pd.1970111002 1800987

[B36] Los FJ van Opstal D Berg C van den Braat AP Verhoef S Wesby-van Swaay E Ouweland AM van den Halley DJ Uniparental disomy with and without confined placental mosaicism: a model for trisomic zygote rescue Prenat Diagn 1998 18 659 668 10.1002/(SICI)1097-0223(199807)18:7<659::AID-PD317>3.0.CO;2-K 9706646

[B37] Felka T Lemke J Lemke C Michel S Liehr T Claussen U DNA degradation during maturation of erythrocytes - molecular cytogenetic characterization of Howell-Jolly bodies Cytogenet Genome Res 2007 119 2 8 10.1159/000109611 18160774

[B38] Vialard F Molina-Gomes D Quarello E Leroy B Ville Y Selva J Partial chromosome deletion: a new trisomy rescue mechanism? Fetal Diagn Ther 2009 25 111 114 10.1159/000203400 19246929

[B39] Iourov IY Vorsanova SG Yurov YB Developmental neural chromosome instability as a possible cause of childhood brain cancers Med Hypotheses 2009 72 615 616 10.1016/j.mehy.2008.12.003 19136219

[B40] Wu TF Chu DS Epigenetic processes implemented during spermatogenesis distinguish the paternal pronucleus in the embryo Reprod Biomed Online 2008 16 13 22 18252043 10.1016/s1472-6483(10)60552-4 PMC2875117

[B41] Migeon BR Jeppesen P Torchia BS Fu S Dunn MA Axelman J Schmeckpeper BJ Fantes J Zori RT Driscoll DJ Lack of X inactivation associated with maternal X isodisomy: evidence for a counting mechanism prior to X inactivation during human embryogenesis Am J Hum Genet 1996 58 161 170 8554052 PMC1914932

[B42] Liehr T Mrasek K Weise A Dufke A Rodríguez L Martínez Guardia N Sanchís A Vermeesch JR Ramel C Polityko A Haas OA Anderson J Claussen U von Eggeling F Starke H Small supernumerary marker chromosomes--progress towards a genotype-phenotype correlation Cytogenet Genome Res 2006 112 23 34 10.1159/000087510 16276087

[B43] Manvelyan M Kempf P Weise A Mrasek K Heller A Lier A Höffken K Fricke HJ Sayer HG Liehr T Mkrtchyan H Preferred co-localization of chromosome 8 and 21 in myeloid bone marrow cells detected by three dimensional molecular cytogenetics Int J Mol Med 2009 24 335 341 19639225 10.3892/ijmm_00000237

[B44] Iourov IY Liehr T Vorsanova SG Yurov YB Interphase chromosome-specific multicolor banding (ICS-MCB): a new tool for analysis of interphase chromosomes in their integrity Biomol Eng 2007 24 415 417 10.1016/j.bioeng.2007.05.003 17627882

[B45] Manvelyan M Hunstig F Bhatt S Mrasek K Pellestor F Weise A Simonyan I Aroutiounian R Liehr T Chromosome distribution in human sperm - a 3D multicolor banding-study Mol Cytogenet 2008 1 25 10.1186/1755-8166-1-9 19014589 PMC2613144

[B46] Yurov YB Iourov IY Vorsanova SG Liehr T Kolotii AD Kutsev SI Pellestor F Beresheva AK Demidova IA Kravets VS Monakhov VV Soloviev IV Aneuploidy and confined chromosomal mosaicism in the developing human brain PLoS One 2007 2 e558 10.1371/journal.pone.0000558 17593959 PMC1891435

